# Challenges and Revisions in Diagnostic Criteria: Advancing Early Detection of Prion Diseases

**DOI:** 10.3390/ijms26052037

**Published:** 2025-02-26

**Authors:** Mika Inada Shimamura, Katsuya Satoh

**Affiliations:** 1 Biomedical Research Support Center, Nagasaki University, 1-12-4 Sakamoto, Nagasaki 852-8523, Japan; shima-m@nagasaki-u.ac.jp; 2 Unit of Medical and Dental Sciences, Department of Health Sciences, Nagasaki University Graduate School of Biomedical Sciences, Nagasaki 852-8501, Japan; 3 Leading Medical Research Core Unit, Department of Brain Research Unit, Nagasaki University Graduate School of Biomedical Sciences, Nagasaki 852-8523, Japan

**Keywords:** humans, 14-3-3 proteins, brain, biomarkers, early diagnosis

## Abstract

Prion diseases are fatal neurological disorders characterized by abnormal protein accumulation in the brain, leading to neurodegeneration, dementia, and ataxia. Sporadic Creutzfeldt–Jakob disease (sCJD), the most common form, accounts for 80–90% of cases and progresses rapidly, with most patients surviving <6 months to a year after symptom onset, indicating the importance of early diagnosis. The disease is classified into six subtypes based on PRNP gene polymorphisms, with differences in protein degradation patterns contributing to the diversity of clinical symptoms. However, diagnosis remains challenging because of the variability in clinical presentation and disease duration. Traditional diagnostic criteria established by the World Health Organization (WHO) rely on clinical findings, electroencephalogram, and cerebrospinal fluid tests, such as the 14-3-3 protein assay. However, these criteria require pathological confirmation, often delaying diagnosis. The recently proposed Hermann’s criteria represent a significant advancement by incorporating newer biomarkers, including magnetic resonance imaging, real-time quaking-induced conversion assay, tau protein, and neurofilament light chain. These criteria improve diagnostic sensitivity and specificity but have a slightly higher risk of false positives. This review compares the effectiveness of these biomarkers with the WHO criteria and highlights the importance of early diagnosis for improving patient care.

## 1. Introduction

Prion disease is a fatal disorder characterized by the accumulation of abnormal protein in the brain, leading to neuronal degeneration and symptoms such as dementia and ataxia. The disease arises when normal prion protein (PrP^C^), primarily composed of α-helix structures, transforms into its abnormal form (PrP^Sc^), which has a higher β-sheet content in the central nervous system [1,2]. In normal prion proteins, approximately 40% of the structure consists of α-helix content, while 3–10% is β-sheet content. Conversely, in abnormal prion proteins, the α-helix content decreases to 20–30%, while the β-sheet content increases by 40–50%, significantly altering their secondary structure. These structural changes drive the abnormal aggregation of prion proteins and their neurotoxicity.

Human prion diseases are classified into three types based on their cause: sporadic, genetic, and acquired. Sporadic Creutzfeldt–Jakob disease (sCJD) accounts for 80–90% of cases and progresses rapidly, often leading to an immobile and mute state within months, underscoring the importance of prompt diagnosis [3].

The symptoms of prion diseases evolve over three stages. In the early stage, patients may experience memory loss, mood disturbances, fatigue, and visual abnormalities such as blurred vision. The middle stage is characterized by pronounced neurological symptoms, including cognitive decline (such as impaired judgment in daily life), motor impairment (such as muscle stiffness, difficulty walking, and lightheadedness), language impairment (such as difficulty speaking), and sensory distortions (such as illusions and hallucinations). In the late stage, independent living becomes impossible due to severe impairments, including loss of consciousness, muscle rigidity, limb immobility, loss of certain reflexes (such as the patellar reflex), and generalized weakness due to weight and muscle loss. Eventually, respiratory failure leads to death. With the rapid progression of symptoms, often within six months to a year, early diagnosis is critical [4]. Symptomatic treatment to alleviate suffering and care strategies aimed at improving quality of life are essential.

sCJD is classified into several subtypes based on a combination of the *PRNP* gene and prion protein degradation patterns. sCJD can be categorized as MM type, MV type, and VV type according to the polymorphism at codon 129 of the *PRNP* gene, involving methionine (M) to valine (V) changes. Additionally, PrP^Sc^ are classified into two types: Type 1 and Type 2, based on their fragment size. The combination of these genetic and protein features yields six subtypes: MM1, MM2, MV1, MV2, VV1, and VV2 [5].

Since the duration and clinical presentation of sCJD vary by subtype, diagnosis can be challenging. Advances in imaging techniques and cerebrospinal fluid (CSF) biomarkers have facilitated more accurate and timely diagnoses.

## 2. CSF Biomarkers

### 2.1. The 14-3-3 Proteins

In CJD, rapid neuronal degeneration leads to the leakage of 14-3-3 protein into the CSF. The importance of 14-3-3 protein was first reported in the 1980s, and its physiological roles, such as the regulation of phosphoproteins [6,7], cell cycle control [8], and inhibition of apoptosis [9,10,11] were elucidated in the 1990s.

In 1996, Hsich et al. assessed the relationship between neurological diseases and 14-3-3 protein using two-dimensional electrophoresis of brain tissue from sCJD patients and controls. They identified 14-3-3 protein as specific to the brain tissue and CSF of CJD patients [12]. That same year, Zerr et al. analyzed CSF samples from 289 prion disease patients, finding 14-3-3 protein positivity in 89.5% (155/173) of genetic CJD (gCJD) cases, demonstrating its diagnostic utility [13].

The diagnostic criteria for prion diseases have evolved over time. In 1996, Masters et al. proposed criteria that categorized diagnoses into three categories—Definite, Probable, and Possible—based on clinical findings, electroencephalography (EEG) and CSF biomarkers such as 14-3-3 protein [14]. However, pathological confirmation via brain biopsy or autopsy was required for a definitive diagnosis, often limiting initial diagnosis to the postmortem stage. To unify diagnostic standards, the WHO introduced criteria in 1998 for various prion diseases, including sCJD, variant CJD (vCJD), and gCJD [15].

Although 14-3-3 protein detection via Western blot (WB) was widely adopted, it showed limited sensitivity, significant data variability, and was time-consuming and costly due to its complex protocol [16]. Therefore, there was an urgent need for standardized assays and quantitative criteria for 14-3-3 protein detection.

To address this, we developed an Enzyme-Linked Immunosorbent Assay (ELISA) kit for detecting 14-3-3γ protein in 2011. Initially, the ELISA kit demonstrated a cutoff value of >6830 AU/mL, with a sensitivity of 95.2% and a specificity of 72.7%. Subsequent re-analysis with collaborators refined the cutoff to >20,000 AU/mL, improving sensitivity to 88% and specificity to 96%. Moreover, WB results were consistent with ELISA findings, with trace 14-3-3 protein WB reactions corresponding to 19,015–23,630 AU/mL and weak WB reactions aligning with 37,188–48,123 AU/mL [17].

Many researchers have adopted the 14-3-3γ ELISA in recent studies, achieving our goal of standardizing the detection of 14-3-3 proteins. The sensitivity and specificity of the 14-3-3γ ELISA range between 80% and 90% and 70% and 92%, respectively, enabling consistent comparison of values across countries [18,19].

### 2.2. Total Tau (t-tau) Protein

The importance of CSF total tau (t-tau) protein levels in patients with human prion diseases (HPD) has been well established. Otto et al. (2002) reported significantly elevated t-tau levels in HPD patients compared to those with other neurological disorders [20]. Subsequently, Riemenschneider et al. established a diagnostic cutoff value of >1300 pg/mL, demonstrating a sensitivity of 80–90% and a specificity of 80–92% [21].

Recent studies have investigated specific tau isoforms in the CSF. Chen et al. showed that antibodies targeting peptides encoded by exons 2, 3, and 10 of the human tau protein could effectively characterize tau profiles in CSF samples from patients with probable CJD [22].

Since 2000, the commercial availability of tau-related ELISA kits has grown significantly, with more than 10 manufacturers now providing these diagnostic tools. However, this expansion has introduced challenges in standardization, as different kits often establish unique cutoff values. The use of various kits across countries complicates international comparisons of t-tau levels in HPD patients [17].

### 2.3. Real-Time Quaking-Induced Conversion (RT-QuIC) Assay

In recent years, several biomarkers have been developed, emphasizing their importance in diagnostic testing. Among these, diffusion-weighted imaging (DWI) and real-time quaking-induced conversion (RT-QuIC) assays have emerged as valuable tools for early diagnosis. The RT-QuIC method uses aberrant Prion Protein (PrP) as the nucleus (seed) for an amplification reaction, detecting abnormal PrP in CSF samples by inducing recombinant PrP (rPrP) aggregation and fibril formation in vitro.

To optimize the assay’s sensitivity and minimize false-positive results, conditions were carefully refined to suppress spontaneous fibril formation without suppressing seed (abnormal PrP)-dependent reactions. This breakthrough was achieved after extensive trial and error. The real-time aspect of the RT-QuIC assay is enabled by monitoring the aggregation process through the fluorescence intensity of thioflavin T, a dye that specifically binds to amyloid fibrils and emits fluorescence. This allows for a simple, high-throughput, and real-time detection system capable of processing a large number of samples.

Additionally, the CSF RT-QuIC assay has demonstrated significant diagnostic potential for sCJD, with sensitivity rates consistently reported between 80% and 100% [23]. In recognition of its clinical utility, the European CJD Surveillance Network incorporated RT-QuIC into its diagnostic criteria for sCJD in 2017 [24].

However, critical nuances exist in the diagnostic performance of RT-QuIC, as sensitivity varies significantly depending on the molecular subtype of sCJD. Studies by Peden et al. revealed critical limitations of the assay, particularly for specific subtypes. For example, the VV1 and MM2 subtypes exhibit markedly reduced sensitivity, posing challenges for comprehensive diagnostic strategies [25,26].

These findings highlight the complexity of prion disease diagnostics and emphasize the importance of considering molecular heterogeneity when interpreting RT-QuIC results. Future research should focus on elucidating the underlying causes of these variations in sensitivity and on developing improved diagnostic approaches to address these limitations.

The specificity of CSF RT-QuIC is remarkably high, reported to be nearly 100%, indicating its ability to specifically detect the presence of PrPSc. However, rare false positives have been reported, with rates around 1% to 2%. These false positives are often attributed to samples from individuals with epilepsy or Alzheimer’s disease [27].

### 2.4. Neurofilament Light Chain (NfL)

Neuronal damage and loss are key pathological mechanisms underlying permanent disability in a wide range of acute and chronic neurological disorders [28]. Within this context, the neurofilament light chain (NfL) has emerged as a crucial biomarker for neurodegeneration. NfL, along with medium and heavy neurofilament chains, forms essential structural components of the neuronal cytoskeleton. When neuronal or axonal damage occurs, these proteins are released into the CSF and subsequently into the bloodstream, making NfL an accessible and valuable marker of neurological injury [29].

In neurodegenerative diseases such as amyotrophic lateral sclerosis, dementia with Lewy bodies, and Parkinson’s disease, NfL proteins form distinctive liquid crystal gel networks. The formation and characteristics of these networks are intricately regulated by the stoichiometric relationships between subunits and their phosphorylation states, adding a layer of complexity to their role in disease pathogenesis [30,31,32].

The evolution of NfL detection methods has dramatically improved our ability to monitor these proteins. Early detection techniques, including first-generation immunoblots and second-generation ELISA methods, were limited in sensitivity, detecting only substantial changes in NfL levels [28]. However, advances in technology have led to the development of third-generation electrochemiluminescence assays and fourth-generation single-molecule array techniques. These newer methods provide exceptional sensitivity, allowing for the reliable detection of even subtle changes in NfL levels. This capacity extends to patients with mild disease and even healthy individuals.

These technological breakthroughs have facilitated numerous multicenter studies exploring the potential of NfL as a biomarker of brain tissue damage. The implications are promising, particularly for longitudinal disease monitoring and therapeutic drug trials, where precise and early detection of neurodegeneration can guide clinical decisions and treatment strategies.

Particularly in the context of sCJD, multiple studies have established NfL as a highly reliable diagnostic marker for distinguishing affected patients from healthy controls [33]. In CSF samples, NfL demonstrates impressive diagnostic accuracy, with sensitivity ranging from 90% to 96% and specificity between 80% and 85% [34,35]. Although serum NfL measurements show slightly different performance characteristics, with sensitivity ranging from 80% to 100% and specificity between 75% and 85% (Kovacs et al., 2017 [36]), they remain robust and clinically valuable.

Building on this compelling evidence, researchers increasingly advocate for NfL as a sensitive and specific blood-based biomarker for diagnosing both genetic and sporadic CJD. Furthermore, NfL has demonstrated potential in predicting disease onset, further enhancing its diagnostic and prognostic utility [37]. A summary of the biomarkers is shown in Table 1.

### 2.5. Magnetic Resonance Imaging (MRI) Biomarkers

MRI is a noninvasive diagnostic tool that imposes less physical burden on patients compared to CSF collection and has significantly simplified the diagnostic process for sCJD. Between 1990 and 2000, MRI technology became widely available, driving rapid advances in its application for diagnosing neurological conditions.

In the late 1990s, abnormal signals on Diffusion-Weighted Image (DWI) were identified as specific indicators for CJD, allowing for early diagnosis without the need for pathological confirmation [38]. By 2000, further improvements in DWI and Fluid-Attenuated Inversion Recovery (FLAIR) imaging allowed for the detection of brain abnormalities specific to CJD with high accuracy. These imaging modalities could identify early disease changes that were undetectable using EEG or CSF tests. Additionally, MRI studies have proven valuable not only in confirming sCJD but also in distinguishing it from clinically similar conditions, highlighting its importance as a diagnostic tool [39,40].

MRI diagnostic criteria for CJD have also evolved over time. Previously, the diagnosis required observing high-signal changes on FLAIR or DWI in two or more areas of the cerebral cortex (excluding the frontal lobe) or in both the caudate nucleus and putamen. However, these criteria had limitations, including low sensitivity at approximately 83%, leading to potential misdiagnoses in cases with atypical findings or early-stage disease [24,41].

To address these challenges, updated MRI diagnostic criteria have been proposed in recent years. The revised criteria state that sCJD can be diagnosed if high-signal-intensity changes are observed on DWI in one or more of seven specific brain regions: the frontal lobe, parietal lobe, occipital lobe, temporal cortex, caudate nucleus, putamen, or thalamus. These updated criteria offer improved diagnostic sensitivity ranging from 90% to 95%, compared to earlier standards [42].

Additionally, conventional MRI diagnostic criteria did not fully account for the diversity of MRI findings associated with molecular subtypes of sCJD. For example, in MM2 cortical-type and VV1-type sCJD, high-signal-intensity changes are typically more pronounced in the cerebral cortex, with relatively minor involvement of the basal ganglia [43,44]. As a result, such cases may not meet the traditional diagnostic criteria for sCJD. In an Australian study, this updated criterion was validated with a sensitivity of 92.3% and a specificity of 85.7%, surpassing the conventional criteria [45].

Meissner et al. further proposed the potential for classifying CJD subtypes based on the distribution of lesions observed on MRI [46], establishing an imaging-based diagnostic approach that detects brain lesions in CJD patients with high accuracy.

The inclusion of MRI in the diagnostic criteria has addressed several challenges, notably enabling earlier diagnosis. Previously, a definitive diagnosis relied on pathological evidence of prion disease, such as the detection of abnormal prion protein in brain tissue, spongiform degeneration, neuronal loss, glial proliferation, or abnormal proteins in immunohistochemistry. The inclusion of MRI has significantly improved diagnostic sensitivity and specificity, facilitating the early identification of gCJD by the detection of presymptomatic lesions and allowing diagnosis even when EEG and CSF findings are negative. Combining MRI findings with CSF biomarkers is expected to further enhance diagnostic accuracy.

### 2.6. The Combination of Biomarkers

In the diagnosis of prion diseases, combining multiple biomarkers has shown promise in improving diagnostic accuracy. MM1 cases, which account for approximately 70% of sCJD cases [47], progress rapidly, often within weeks. By the time typical symptoms meet the initial WHO criteria, the disease is usually at an advanced stage. In the absence of effective treatments, early diagnosis is the only viable approach for disease prevention. Moreover, even if innovative therapies are developed, starting treatment after a definitive diagnosis may be too late for meaningful outcomes. This highlights the importance of achieving a rapid and accurate diagnosis at the earliest stage of disease progression.

The RT-QuIC assay is a highly sensitive method for detecting PrP^Sc^ in the CSF. While the RT-QuIC assay alone shows high diagnostic accuracy, combining it with other biomarkers can enhance reliability [48,49]. For example, the combination of 14-3-3 protein, t-tau and neuron-specific enolase with RT-QuIC improves the diagnostic accuracy [15,16,50].

Moreover, MRI findings play an important role in diagnosing sCJD; however, in the early stages or atypical cases, MRI results may be inconclusive. Combining MRI findings with CSF biomarkers improves diagnostic accuracy. If positive findings are present on both DWI and the RT-QuIC assay, the reliability of the diagnosis increases [50,51]. Therefore, the combination of biomarkers, MRI findings, and clinical assessments is essential for accurate diagnosis.

## 3. Diagnostic Accuracy

Hermann et al. proposed new diagnostic criteria that incorporate advanced biomarkers into the WHO framework, allowing early clinical diagnosis regardless of the disease stage [23]. In a retrospective cohort study, Nonaka et al. compared the validity of Hermann’s criteria with the WHO criteria, focusing on their sensitivity and specificity for diagnosing early sCJD [52]. An important distinction is that the WHO criteria combine clinical symptoms with ancillary tests, whereas Hermann’s criteria prioritize MRI findings. The WHO criteria are well established and include clinical symptoms, EEG, and 14-3-3 protein testing, which are relatively easy to implement but may have limitations in sensitivity. In contrast, Hermann’s criteria, which emphasize biomarkers detectable in the early stages of the disease, enable earlier diagnosis.

Hermann’s criteria showed an excellent sensitivity of 99.3%, compared to the WHO’s 96.4%, indicating an improved ability to correctly identify sCJD cases. However, the specificity was slightly lower (95.2%) than that of the WHO criteria (96.6%), suggesting a higher risk of false positives. While the inclusion of new biomarkers in Hermann’s criteria addresses some of these concerns, the slightly higher risk of false positives means clinical caution is required, particularly when distinguishing sCJD from treatable neurological disorders such as autoimmune encephalitis and epilepsy, which can result in false-positive findings.

Nevertheless, if appropriate exclusionary diagnoses are made, Hermann’s criteria are highly effective for early diagnosis. Specifically, a positive RT-QuIC assay strongly indicates the possibility of sCJD, even in the absence of typical symptoms, facilitating early disease detection. Through careful differential diagnosis and multifaceted evaluation, Hermann’s criteria can significantly contribute to the early diagnosis of sCJD [52]. However, clinicians must apply these criteria carefully to ensure accurate diagnosis and minimize the risk of false positives.

Furthermore, while Hermann’s criteria are specific to sCJD, the WHO criteria are more versatile and applicable to a wider range of prion diseases, making them more suitable for clinical practice. As such, further refinement of the diagnostic criteria will be necessary in the future.

## 4. The Critical Role of Early Diagnosis in Prion Diseases: Current Perspectives and Evolving Diagnostic Approaches

Early diagnosis of prion diseases presents one of the most significant challenges and opportunities in contemporary neurology. While curative treatments are still lacking, timely diagnosis is invaluable for patient care, family support, and research advancement. This review explores the multifaceted importance of early diagnosis, current diagnostic methodologies, and emerging approaches in prion disease detection.

### 4.1. Fundamental Importance of Early Diagnosis

The significance of early diagnosis in prion diseases encompasses several critical aspects. First, it provides patients and their families with precious time to process and adapt to the diagnosis. Unlike many other neurological conditions, prion diseases typically progress rapidly, making this adaptation period particularly crucial. Early diagnosis empowers families to make informed decisions about care preferences, engage in advanced care planning, and maximize quality time with their loved ones.

Furthermore, an accurate early diagnosis helps prevent the physical, emotional, and financial burdens associated with unnecessary investigations and ineffective treatments that might arise from diagnostic uncertainty. This is especially relevant given the diverse presentation of prion diseases and their potential to mimic other neurological conditions.

### 4.2. Therapeutic Implications and Research Perspectives

The therapeutic implications of early diagnosis extend beyond immediate patient care. Parallels with other neurodegenerative conditions, particularly Alzheimer’s disease (AD), provide valuable insights. In AD, the concept of preclinical intervention has gained significant attention, with evidence suggesting that early therapeutic intervention—before significant cognitive decline—may provide the best opportunity for disease modification. Similar principles may apply to prion diseases, emphasizing the critical importance of early detection for future therapeutic trials [53].

Currently, the diagnosis of prion diseases relies primarily on two approaches: advanced neuroimaging and biochemical analysis of biological fluids. Each method offers unique advantages and contributes to overall diagnostic accuracy.

### 4.3. Magnetic Resonance Imaging in Prion Disease Diagnosis

MRI has emerged as a cornerstone in the diagnosis of prion diseases, offering valuable insights into the characteristic imaging patterns that define these conditions. Over time, the criteria for MRI-based diagnosis have evolved, reflecting a deeper understanding of the disease’s pathological features. The conventional diagnostic criteria for prion diseases require the identification of high-signal changes in multiple brain regions. Specifically, these criteria involve either high-signal changes in two or more cortical regions (excluding the frontal lobes) or bilateral involvement of the caudate nucleus and putamen, as demonstrated through DWI.

A modern approach to MRI criteria, as proposed by Bizzi et al. [44], simplifies the diagnostic process by requiring high-signal changes in any one of seven specific brain regions: the frontal lobe, parietal lobe, occipital lobe, temporal lobe, caudate nucleus, putamen, or thalamus. These simplified criteria aim to streamline the diagnostic process while maintaining accuracy.

Comparative analyses of the traditional and modern MRI diagnostic criteria have shown impressive diagnostic accuracy. The traditional MRI criteria showed a sensitivity of 92.3% and a specificity of 87.3%, while the newer proposed criteria demonstrated a sensitivity of 92.3% and a specificity of 85.7% [45]. These findings indicate that the modern criteria, while slightly less specific, offer equivalent sensitivity to the traditional criteria. The simplified modern approach could be advantageous for routine clinical use, providing a more reliable method for diagnosing prion diseases.

Key findings regarding early diagnosis:

1. The simplified criteria maintained diagnostic accuracy (92.3% sensitivity, 85.7% specificity) while being easier to apply, potentially enabling faster diagnosis.
2. Early-stage imaging characteristics:
  - DWI shows hyperintense signals first;
  - Fluid-Attenuated Inversion Recovery (FLAIR) may initially appear normal;
  - ADC maps show iso- to hypointense signals in affected areas.
3. Multimodal imaging importance:
  - DWI is most sensitive for early detection;
  - FLAIR complements DWI and helps rule out other conditions;
  - Combined DWI-FLAIR-ADC approach provides most reliable early diagnosis.
4. Expert application of the criteria significantly outperformed routine diagnostic reports, suggesting the importance of neuroradiologist experience in early detection.

The simplified criteria could lead to earlier diagnosis while maintaining diagnostic accuracy, potentially reducing unnecessary testing and allowing for more timely patient care interventions. The key to successful early diagnosis appears to be the combination of appropriate imaging sequences and experienced interpretation.

### 4.4. RT-QuIC Analysis: A Revolutionary Diagnostic Tool

The RT-QuIC assay has revolutionized the diagnosis of prion disease by providing a highly sensitive method for detecting disease-specific protein misfolding. This assay has demonstrated significant diagnostic utility, as evidenced by comprehensive surveillance data from a large cohort comprising 850 sCJD patients and 400 controls [19]. First-generation RT-QuIC showed an overall sensitivity of 90% and specificity of 99% [48]. First-generation RT-QuIC proved particularly effective in detecting the most common sCJD subtypes, including MM/MV1, VV2, and MV2 [54,55]. Second-generation RT-QuIC (IQ) further enhanced diagnostic capabilities, achieving a sensitivity range of 92% to 96% while maintaining high specificity. This IQ assay also offered improved detection of variant subtypes.

### 4.5. Clinical Applications and Limitations

The practical implementation of diagnostic tools for prion diseases involves a complex interplay of clinical challenges, technical considerations, and interpretative nuances. Understanding these aspects is crucial for both clinicians and researchers. Notably, variability in clinical presentation include a wide range of initial symptoms, atypical presentations that may delay recognition, and overlap with other neurological conditions. Technically, the implementation of diagnostic tools requires standardized protocols to ensure consistency and reliability across different healthcare settings. The importance of quality control measures cannot be overstated, as variations in equipment and expertise across centers can affect diagnostic accuracy.

The interpretation of diagnostic results also presents challenges. Clinicians must integrate multiple diagnostic modalities, a process that can be complicated by conflicting results, as different tools may yield varying degrees of sensitivity and specificity. Additionally, borderline cases where diagnostic criteria are not fully met, require careful handling to avoid misdiagnosis.

### 4.6. Unusual Clinical Presentations of Sporadic CJD

To establish accurate diagnoses of prion diseases at early stage, clinicians must possess comprehensive knowledge that extends beyond typical presentations to encompass unusual variants. Understanding this complete spectrum is crucial, as atypical manifestations may deviate significantly from classical descriptions. This comprehensive diagnostic approach enables more precise identification of cases that might otherwise be overlooked using conventional diagnostic criteria [56].

#### 4.6.1. Visual Disorders (Heidenhain Variant of CJD)

Patients with CJD may present with visual disturbances, known as the Heidenhain variant (HvCJD). This occurs in 3.7–4.9% of sCJD cases. Common symptoms include blurred vision, visual field defects, metamorphopsia, color misperceptions, and visual agnosia. These symptoms arise from dysfunction in occipital and parietal cortices. HvCJD is most commonly associated with MM(V)1 or MM2C (MM2 with predominant cortical pathology) subtypes of sCJD.

#### 4.6.2. Seizures Pattern

Seizures occur in 15–20% of CJD cases, typically late in the disease course and mainly in MM(V)1 subtype. As an initial symptom, seizures are rare (0–3%). They commonly present as epilepsia partialis continua (EPC) or nonconvulsive status epilepticus (NCSE). Treatment with antiepileptic drugs is usually ineffective.

#### 4.6.3. Psychiatric Disorders

Psychiatric symptoms can be the initial presentation of CJD, occurring in 7.4–11% of sCJD cases. These include psychotic features, mood disorders, and agitated behaviors. This presentation is more common in younger adults and often leads to misdiagnosis. Depression is typically resistant to antidepressant treatment.

#### 4.6.4. Movement Disorders

CJD can manifest with various movement disorders. The most notable presentation is corticobasal syndrome (CBS), which manifests as asymmetric rigidity, apraxia, and the distinctive alien limb phenomenon. Some patients develop symptoms resembling progressive supranuclear palsy, though this is very rare, and these patients typically experience vertical gaze palsy along with unexplained falls. Pseudobulbar palsy represents an extremely rare manifestation, occurring in only 0.5% of cases, where patients struggle with speech and swallowing. Additionally, CJD patients may exhibit a range of involuntary movements including myoclonus, dystonia, and choreoathetosis. Understanding these varied movement disorder presentations is crucial for proper diagnosis, as they can sometimes be the initial manifestation of CJD.

#### 4.6.5. Aphasia

While common during disease progression, aphasia as an isolated initial symptom occurs in only 1% of cases. Symptoms typically include naming difficulties and reduced verbal fluency. This presentation is mainly associated with MM(V)1 subtype but can occur in MM2C and MV2K (MV2 with kuru amyloid plaques) subtypes.

#### 4.6.6. Stroke-like Onset Pattern

Some CJD cases present with acute neurological symptoms mimicking stroke, including hemiparesis, visual disturbances, and speech problems. Diagnostic challenges arise because initial CT scans may be unremarkable, and DW-MRI findings can be confused with acute stroke. This presentation is most commonly associated with MM(V)1 subtype

### 4.7. Differentiation Between CJD and Mimics

Creutzfeldt–Jakob disease (CJD) represents a common cause of rapidly progressive dementia (RPD), triggered by the proliferation of abnormal prion proteins. While diagnostic confirmation relies on highly sensitive and disease-specific tests such as RT-QuIC, these assessments may require several days to yield results. The spectrum of conditions mimicking CJD symptoms encompasses autoimmune encephalitis, neurosarcoidosis, frontotemporal lobar degeneration, dural arteriovenous fistula, cerebral amyloid angiopathy, and systemic lupus erythematosus [57].

### 4.8. Diagnostic Indicators for Early Detection

The distinction between CJD and its mimics can be achieved through a combination of clinical features and rapid diagnostic modalities, including MRI, electroencephalography, and routine CSF analysis. Mimicking conditions frequently present with early motor dysfunction, manifesting as faciobrachial dystonic seizures, myoclonus, dyskinesia, and parkinsonism, alongside CSF abnormalities characterized by pleocytosis and elevated protein levels [41].

While RT-QuIC testing typically yields positive results in CJD patients, mimics generally present with negative findings. Total tau protein levels tend to be elevated in CJD patients compared to mimics, though this biomarker’s non-specific nature warrants careful consideration. Additionally, autoantibody testing may prove valuable in establishing a diagnosis of autoimmune encephalitis.

### 4.9. Diagnostic Criteria at Early Stages of sCJD

Early diagnosis of CJD remains challenging when relying solely on current clinical features, MRI, EEG, and routine CSF testing; differentiation from CJD-mimicking disorders plays a crucial role in early diagnostic accuracy.

1. In our clinical experience, early-stage CJD typically presents with minimal symptomatology, manifesting zero to one of the established clinical criteria (rapidly progressive cognitive impairment, myoclonus, visual impairment or cerebellar disturbance, pyramidal or extrapyramidal disorders).
2. CSF analysis reveals characteristic patterns:
  - Minimal to absent pleocytosis;
  - Protein levels within physiological parameters;
  - Modest elevation of total tau protein;
  - Approximately 50% RT-QuIC positivity.
3. Characteristic neuroimaging findings include
  - Restricted diffusion in caudate or caudate/putamen or caudate/putamen/thalamus, or at least two cortical regions (temporal, parietal, occipital) on MRI brain scan, no subcortical white matter involvement, no isolated restricted diffusion in the thalamus. Characteristic hyperintensities may be seen on FLAIR images, but DWI sequences are required to confirm CJD-typical restricted diffusion and corresponding Apparent diffusion coefficient (ADC) hypo–iso intensities;
  - FLAIR isointensities in cortical regions;
  - Basal ganglia demonstrating hyperintensities on both FLAIR and DWI;
  - Notable absence of hyperperfusion on Arterial Spin Labeling (ASL) imaging; presence of significant hyperperfusion suggests alternative diagnoses.

### 4.10. Future Directions and Emerging Technologies

The future of prion disease diagnosis is shaped by ongoing advancements in imaging techniques, novel MRI sequences, and molecular imaging approaches, coupled with artificial intelligence Alzheimer disease-assisted interpretation. Additionally, blood-based biomarkers and novel CSF markers are being explored to offer more accessible and less invasive diagnostic options, while the combination of biomarker panels promises to improve sensitivity and specificity. Alongside these innovations, refining diagnostic algorithms is key to advancing clinical practice. The integration of multiple diagnostic modalities, such as MRI, biomarkers, and RT-QuIC assays, will enable a more comprehensive approach to prion disease detection. Risk stratification techniques and subtype-specific criteria are also under development, aiming to tailor diagnostic methods to different prion disease variants.

### 4.11. Implications for Clinical Practice and Research

Advancement in early diagnostic capabilities for prion diseases hold substantial implications for clinical practice, research, and healthcare systems. Clinically, early diagnosis facilitates improved patient counseling, better resource allocation, and enhanced care planning. In research, it enables earlier patient identification for clinical trials, provides deeper insights into disease progression, and aids in the development of therapeutic targets. From a healthcare system perspective, early diagnostics help optimize resource utilization, improve cost-effectiveness, and highlight the need for specialized training and education.

## 5. Conclusions and Future Perspectives

Early diagnosis of prion diseases remains a critical goal in neurology. While current diagnostic approaches demonstrate impressive accuracy, ongoing refinement and the development of new techniques are essential. The integration of multiple diagnostic modalities, along with heightened awareness of varied disease presentations, provides the best opportunity for early detection. Future advancements will likely focus on enhancing the sensitivity and specificity of diagnostic tools while ensuring their practical application in clinical settings.

This comprehensive approach to early diagnosis not only improves patient care but also establishes a foundation for future therapeutic trials and potential disease-modifying treatments. Continued research and development in this area are critical to advancing our understanding and management of prion diseases.

## Figures and Tables

**Table 1:** Major Biomarkers for Prion Diseases.

Biomarker	Sample Type	Features	Sensitivity (%)	Specificity (%)
14-3-3 Protein	CSF	Marker of acute neurodegeneration. Elevated in Creutzfeldt–Jakob disease (CJD). Less sensitive and specific than RT-QuIC but useful as a supplementary diagnostic tool.	80–90	70–92
RT-QuIC	CSF	Gold standard for prion disease diagnosis. Highly sensitive and specific.	90–100	95–100
Neuron-Specific Enolase (NSE)	CSF, Blood	Increases as CJD progresses. Also elevated in other neurodegenerative diseases, reducing specificity.	65–85	50–75
Tau Protein	CSF	Indicator of neuronal damage. Also elevated in Alzheimer’s disease, reducing specificity.	85–95	50–80
α-Synuclein	CSF	Useful for differentiating CJD from Lewy body dementia (DLB) and Parkinson’s disease (PD).	65–85	50–75
Neurofilament Light Chain (NfL)	CSF, Serum	Elevated in various neurodegenerative diseases. Also high in ALS, FTD, and other conditions. Can be measured in blood.	85–95	60–85

Abbreviations: CJD, Creutzfeldt–Jakob disease; CSF, cerebrospinal fluid; RT-QuIC, real-time quaking-induced conversion.

## Data Availability

The data were obtained from the patient’s attending physician and the Japan Prion Disease Surveillance Committee at the time of the investigation and pathological autopsy.
